# Observation of a High-Energy Tamm Plasmon State in the Near-IR Region

**DOI:** 10.1021/acsami.2c00486

**Published:** 2022-03-09

**Authors:** Oleksandr Buchnev, Alexandr Belosludtsev, Vassili A. Fedotov

**Affiliations:** † Optoelectronics Research Centre, University of Southampton, Southampton SO17 1BJ, U.K.; ‡ Centre for Photonic Metamaterials, University of Southampton, Southampton SO17 1BJ, U.K.; § Optical Coating Laboratory, Center for Physical Sciences and Technology, Vilnius LT-02300, Lithuania

**Keywords:** Tamm plasmon, nanograting, effective medium approach, hybrid optical cavity, distributed Bragg reflector

## Abstract

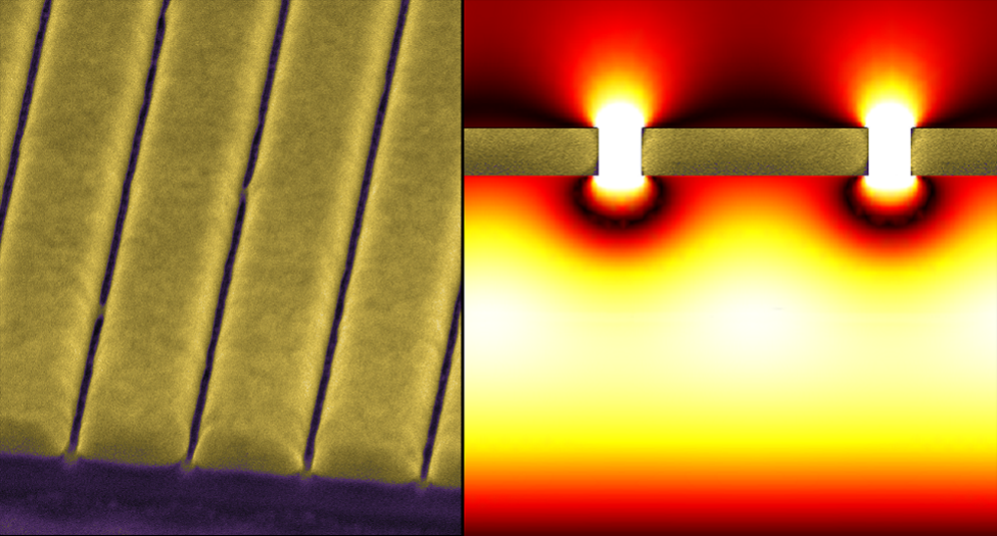

We report on the experimental observation of a Tamm plasmon state in the near-IR region characterized by an anomalously high energy level located in the upper half of the photonic band gap. Such a “blue” Tamm plasmon was demonstrated at the interface between a conventional, completely periodic Bragg reflector and a nanostructured nonresonant thin gold grating. We study the effect of the grating period on the characteristics of the anomalous state and show that the anomaly results from a nontrivial topology of the nanograting’s optical near field, which cannot be captured by the effective medium approach and transfer matrix method commonly employed in the analysis of Tamm plasmons.

## Introduction

In solid-state physics, the breakdown of periodicity occurring at the surface of crystals results in the formation of localized electronic surface states, known as Tamm states.^1^ Kaliteevski and co-workers have recently pointed out that there must be an optical analogue of such states, the so-called Tamm plasmons, which correspond to optical fields confined at the surface of one-dimensional photonic crystals (i.e., Bragg reflectors) terminated by metal layers.^2^ Tamm plasmons have been experimentally demonstrated in ref (3) and are currently regarded as a viable alternative to conventional surface plasmons in many applications, including optical switches,^4^ semiconductor lasers,^5,6^ light emission and harmonics generation,^7−9^ solar cells,^10^ and sensors.^11−17^

One of the advantages of Tamm plasmons over the surface plasmons is that they can also be excited with TE-polarized light (and for any angle of incidence) because their dispersion lies completely within the light cone.^2^ Also, unlike conventional surface plasmons, Tamm plasmons are largely insensitive to ohmic losses in metal layers.^18^ However, the robust nature of Tamm plasmons takes its toll by limiting the range of energies available to these optical surface states. Indeed, since Tamm plasmons reside predominantly inside Bragg reflectors, their energies are determined mainly by the structure of the latter. In particular, for a conventional, completely periodic Bragg reflector, Tamm plasmons are bounded to the lower half of its main photonic band gap.^2^ The existing approaches that allow one to control the energy of Tamm plasmons without altering the structure of Bragg reflectors rely on illumination at oblique incidence,^2,19^ varying metal thickness,^20^ micropatterning of metal layers,^18,21^ or replacing the latter with tunable metasurfaces.^22^ None of these approaches, however, enable the demonstration of Tamm plasmon states above the Bragg energy in periodic Bragg reflectors. It has been recently suggested and shown theoretically that such optical states could appear if Bragg reflectors were interfaced with subwavelength thin-metal nanogratings.^23^

Here, we report on the first experimental demonstration of the predicted high-energy Tamm plasmon state in the near-IR region and provide an insight into its microscopic nature. We also show that the appearance of such a state cannot be described in the frame of the commonly employed transfer matrix method and the effective medium approach, suggesting that the latter has limited applicability to nanostructured nonresonant metal films.

## Fabrication and Characterization

**Figure 1:**
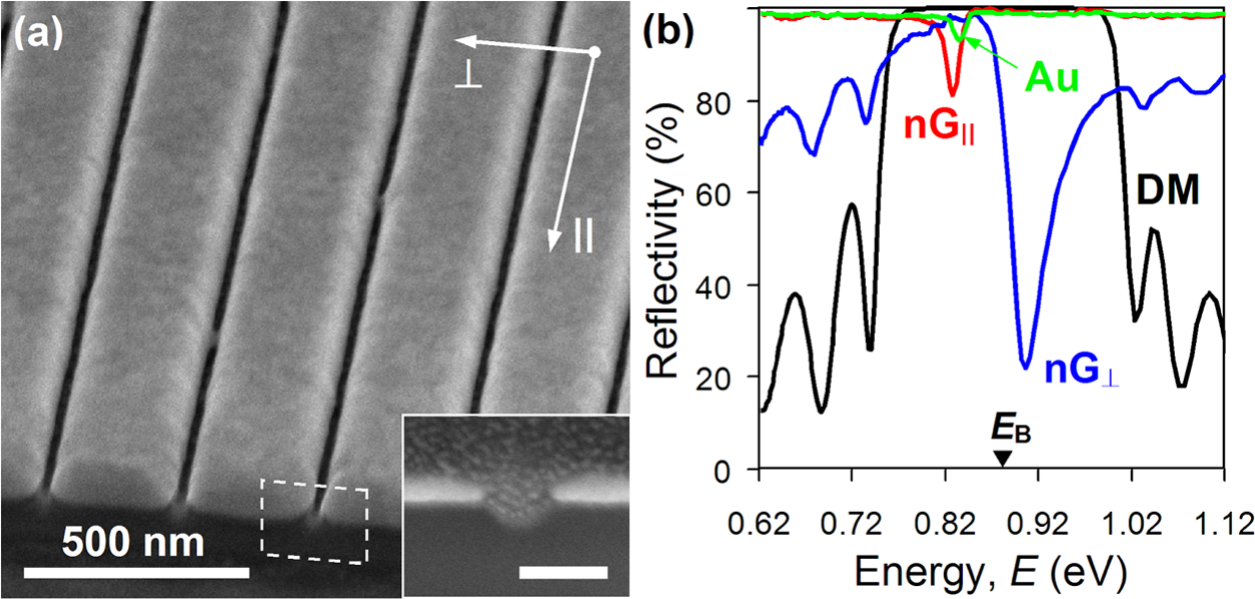
(a) SEM image of a fragment of Au nanograting with the period of 300 nm fabricated on top of a dielectric mirror. Arrows show how the directions along (||) and across (⊥) the slits of the nanograting are defined. The inset shows high-resolution cross section of the nanograting indicated in the main image by a dashed box. Scale bar is 100 nm. Uniform gray in the bottom half is Nb_2_O_5_, white that in the middle is Au, and grainy pattern in the top half corresponds to Pt, which was deposited locally on top of the nanograting to visualize the plane of the cross section and enhance material contrast. (b) Reflectivity spectra measured in different areas of the sample corresponding to a pristine dielectric mirror (black, DM), mirror coated by a continuous 50 nm thick Au film (green, Au), and mirror terminated by the nanograting with its slits being parallel (red, nG_∥_) and orthogonal (blue, nG_⊥_) to incident polarization.

The Bragg reflector in our study was a dielectric mirror, designed to exhibit a 270 meV wide photonic band gap centered at 870 meV (Bragg energy, *E*_B_). It comprised 11 pairs of Nb_2_O_5_ (high index) and SiO_2_ (low index) layers with a thickness of, respectively, 159 and 246 nm, which were deposited onto a 1 in. diameter and 1 mm thick double-side polished fused silica substrate using reactive magnetron sputtering, as detailed in ref (24). A section of the high-index side of the mirror (that is terminated by Nb_2_O_5_ layer) was coated by a 50 nm thick film of Au via thermal evaporation (see the Supporting Information). The deposition of Au film was carried out at room temperature with the rate of about 12 nm/min under a working pressure of 3.5 × 10^–6^ Torr using high-purity Au pellets (purity 99.999%). The nanograting was milled in a 30 μm × 30 μm larger patch of the film using a focused ion beam with the area dosage set to 7 mC/cm^2^ and an ion current of 26 pA (Figure 1a). The slits of the nanograting were cut through the film and had a width of about 50 nm (see the inset to Figure 1a). The period of the pattern was 300 nm, which ensured that the nanograting would operate in the near-IR region as a nonresonant, optically uniform anisotropic film transmitting and reflecting light without diffraction in the substrate and air.

The spectral response of the fabricated sample was characterized in reflection at normal incidence using an imaging microspectrophotometer (CRAIC Technologies) equipped with a tungsten–halogen light source, a broad band linear polarizer, and a cooled near-IR CCD array. Light was focused onto the sample from the Au-coated side of the mirror to a spot of about 1.5 mm in diameter using a 15× objective with NA = 0.28 and collected upon reflection using the same objective. The spectra of the nanograting were acquired for the polarization of incident light set parallel (||) and orthogonal (⊥) to the slits, as indicated in Figure 1a. The spectral measurements were confined to a 22 μm × 22 μm area on the surface of the nanograting, as set by a square aperture installed in the image plane of the instrument. This enabled us to eliminate the contribution from light diffracted off the edges of the nanograting to the measured spectra.

## Results and Discussion

Figure 1b compares the reflectivity spectra acquired in three different areas of our sample, corresponding to pristine and Au-coated sections of the dielectric mirror, and the mirror capped by the nanograting. The spectrum of the pristine mirror confirms the appearance of a photonic band gap typical for Bragg reflectors, which stretches from about 747 to 1015 meV, as prescribed by the mirror’s design. A small (∼10 meV) blueshift of the band gap is attributed to strong angular dispersion common in Bragg reflectors, which our sample exhibits due to focused illumination. In the spectrum of the Au-coated section, this band gap is masked by naturally high and broad-band reflectivity of the metal layer, while a shallow dip visible in the lower half of the band gap signifies the appearance of a conventional Tamm plasmon with the energy of 836 meV (green curve in Figure 1b). The latter is dampened, as expected in the case of a continuous Au film thicker than 30 nm.^20^ Also, its spectral line is broadened and additionally weakened via angular dispersion caused by focused illumination. A similar reflectivity spectrum is displayed by the dielectric mirror capped with the nanograting for incident light polarized along the slits (||), featuring a slightly stronger Tamm plasmon resonance, which is shifted to lower energies by about 8 meV (red curve in Figure 1b).

For the orthogonal polarization (⊥), the spectrum of the nanograting also indicates the appearance of a Tamm plasmon-like state but at a substantially higher energy, namely, 906 meV, which exceeds the Bragg energy by about 25 meV (blue curve in Figure 1b). We regard such a state as anomalous because the highest possible Tamm plasmon energy is attained for a semi-infinite metal layer^2^ and, as we show below, cannot exceed the Bragg energy in our case. Also, given the subwavelength period of its pattern (<λ/4), the nanograting must have effectively behaved as a nonresonant continuous film with metallic response somewhat weaker than that of Au film it was milled in. Hence, intuitively, the phase change acquired by light reflected off the nanograting into the dielectric mirror should be further away from the saturation (which occurs for a semi-infinite Au layer)^25^ and resulted in a Tamm plasmon state with the energy smaller than 836 meV—just like in the other case.

To further demonstrate the anomalous character of the observed high-energy (“blue”) state, we resorted to the well-established transfer matrix method (TMM) commonly employed in the analysis of Tamm plasmons. Using TMM, we calculated (and compare below) the reflectivity spectra of the dielectric mirror covered by the nanograting for incident light polarized along and across the slits. In each case, the nanograting was defined as a 50 nm thick continuous film with the optical constants given by the effective medium approach. To avoid limitations and shortfalls of the existing analytical effective medium models (which could lead to additional artifacts)^26^ we elected to extract the effective optical constants of the nanograting directly from its own transmission and reflection coefficients—just as it was done for thin-metal films.^27,28^ In our case, the transmission and reflection data for the effective medium calculations were obtained numerically, with the help of commercial simulation software COMSOL Multiphysics. Correspondingly, the simulations involved just the nanograting and took into account the effect of only the mirror’s top high-index (i.e., Nb_2_O_5_) layer, which acted as a substrate and was extended to infinity to simplify the extraction of the effective parameters in our model. The optical constants of Au were defined by the tabulated data;^29^ the refractive index of Nb_2_O_5_ was assumed to be constant across the entire energy band and equal to 2.24.^24^ The effective optical constants of the nanograting were given by the values that enabled us to reproduce, using the standard thin-film formulae,^30^ the simulated transmittance and reflectance of the nanograting with the accuracy exceeding 0.3% (see the Supporting Information).

**Figure 2:**
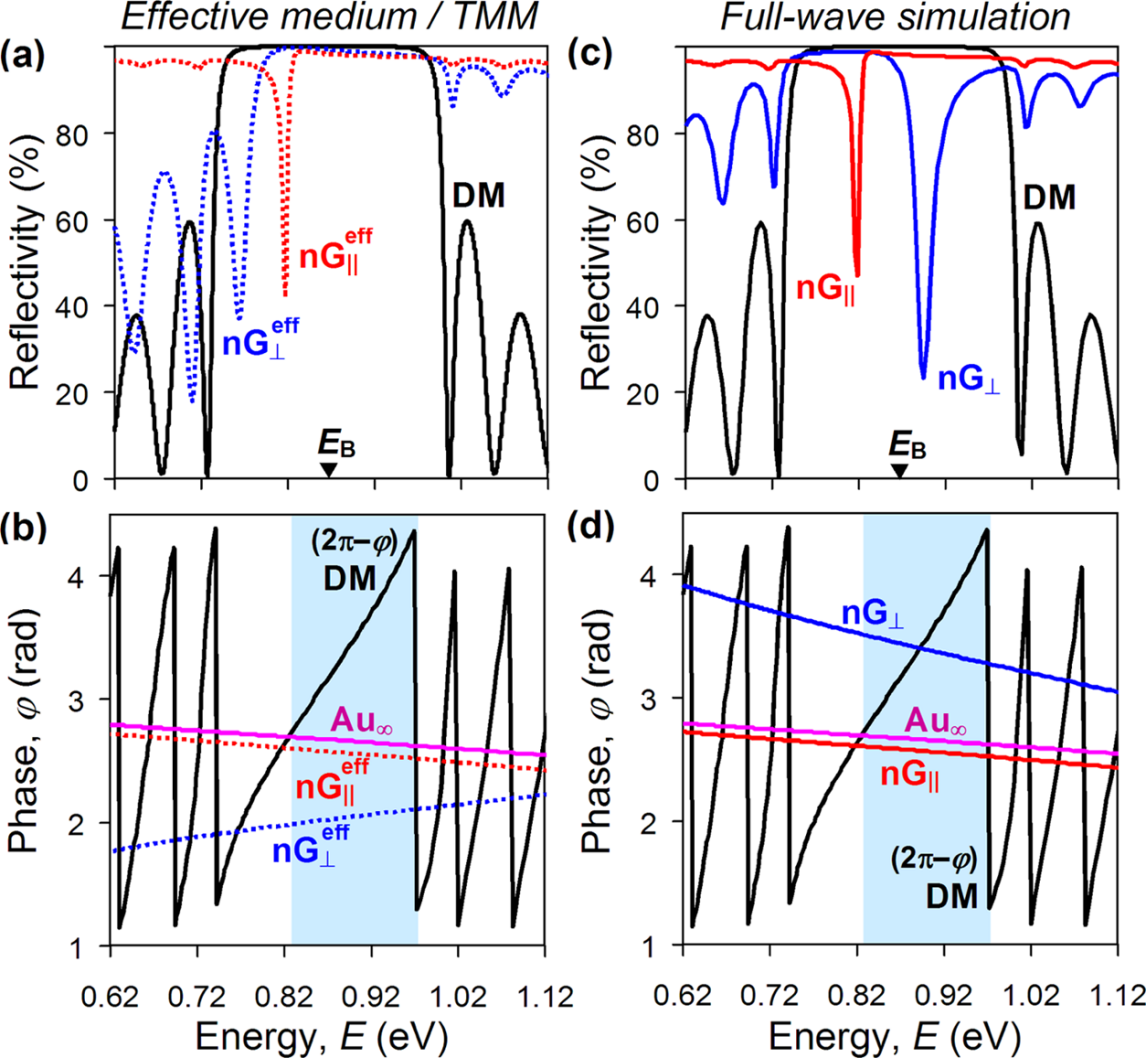
(a) Calculated reflectivity spectra of a pristine dielectric mirror (black, DM) and mirror terminated by Au nanograting with the period of 300 nm and its slits being parallel (dotted red, nG_∥_^eff^) and orthogonal (dotted blue, nG_⊥_^eff^) to incident polarization. The spectra were obtained using TMM with the optical properties of the nanograting fed by the effective medium approach. (b) Calculated dispersions of the reflectivity phase of a pristine dielectric mirror (black, DM), continuous semi-infinite Au layer (magenta, Au_∞_), and the nanograting with its slits being parallel (dotted red, nG_∥_^eff^) and orthogonal (dotted blue, nG_⊥_^eff^) to incident polarization. The reflectivity phase of the nanograting was obtained using the thin-film formula with the optical properties of the nanograting fed by the effective medium approach. The blue shaded region marks the energy band inaccessible to conventional Tamm plasmons. (c) Same as (a) but computed using a full-wave numerical model. (d) Same as (b) but computed using a full-wave numerical model.

The reflectivity spectra of the dielectric mirror capped by the nanograting, as calculated using TMM, are shown in Figure 2a. Clearly, for the polarization of incident light parallel to the slits, the obtained spectrum agrees very well with the experimental data, confirming the appearance of a usual Tamm plasmon resonance at around 818 meV. The slight mismatch between the theory and experiment here in terms of the energy levels (of about 10 meV) results from the strong angular dispersion of the dielectric mirror, which, as we noted earlier, blue-shifts the measured spectrum under focused illumination. For the orthogonal polarization, however, the theory and experiment strongly disagree—TMM calculations predict a dip in reflection at a substantially lower energy, namely, 765 meV. As a further check, we also analyzed the phase matching in both cases since it effectively controls the formation of Tamm plasmon states. In the context of Tamm plasmons, the phase matching ensures that the round trip for light bouncing inside a cavity created by a Bragg reflector and metal film is constructive. For the main photonic band gap of our mirror, the phase matching condition dictates that the total phase change acquired by light upon completing one round trip should be equal to 2π, that is, φ_DM_ + φ_nG_ = 2π,^2,31^ where φ_DM_ and φ_nG_ are changes of the phase caused by reflections off the dielectric mirror and nanograting (into the dielectric), respectively. The energy at which this equality holds can be found graphically as the point of intersection between [2π – φ_DM_(*E*)] and φ_nG_(*E*) curves. The former is given by TMM, while the latter is readily extracted from the thin formula after plugging in the effective optical constants of the nanograting. As evident from Figure 2b, the phase matching for both polarizations is achieved at the energies of the reflectivity dips predicted by our TMM calculations. We further note that in the limiting case of a semi-infinite metal layer, the phase matching occurs at a higher energy, 825 meV (intersection of black and magenta curves in Figure 2b), though still well below Bragg energy. Correspondingly, the energy band above 825 meV must remain seemingly inaccessible to Tamm plasmons (blue shaded region in Figure 2b).

**Figure 3:**
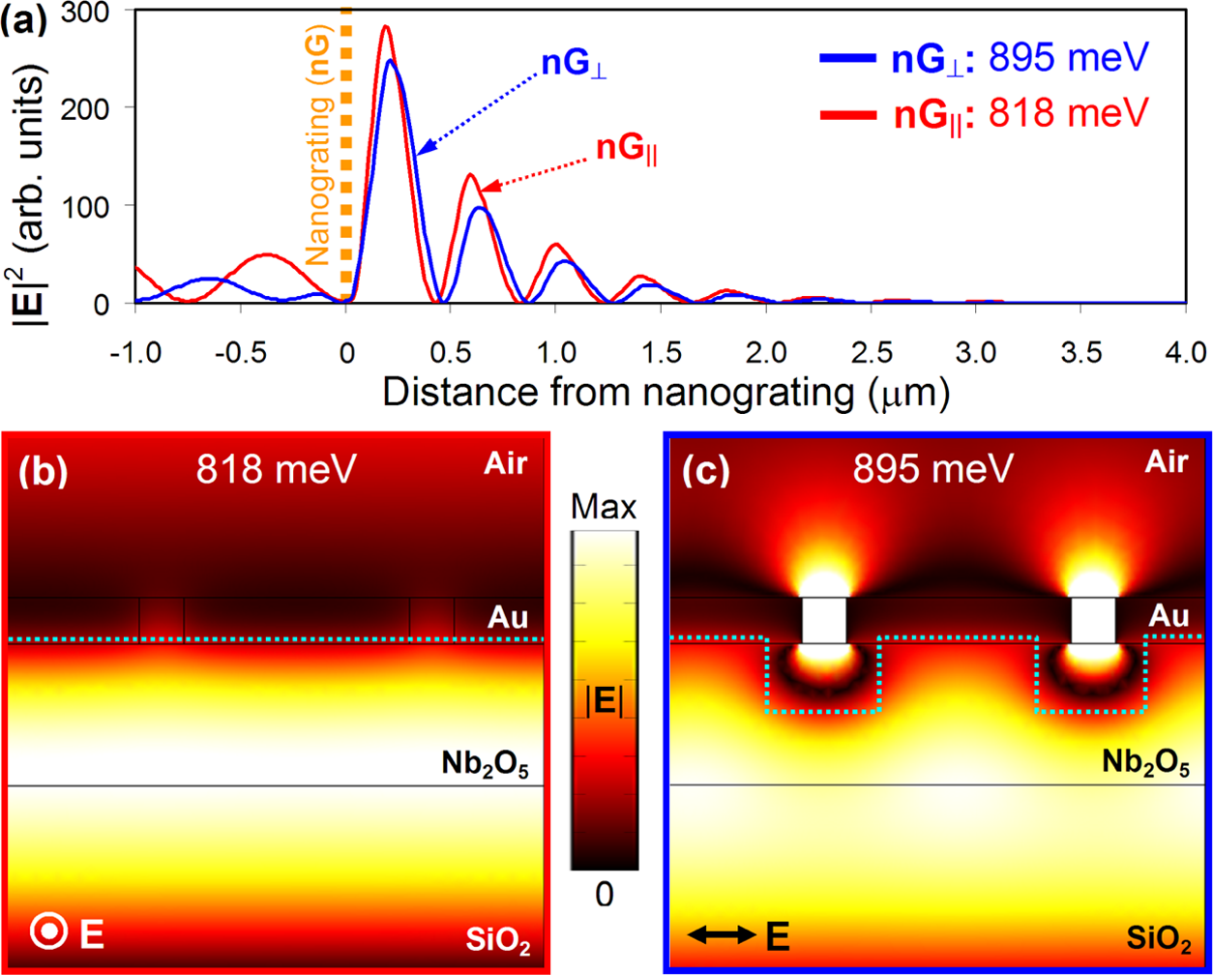
(a) Electric field intensity as a function of distance from Au nanograting (nG) in a dielectric mirror (positive axis) and air (negative axis) calculated at 818 meV (red) and 895 meV (blue) resonances visible in Figure 2c. (b) Distribution of the electric field amplitude near the surface of Au nanograting with the period of 300 nm (in the plane orthogonal to its slits) calculated at 818 meV resonance. The light blue dotted line marks the effective borderline between the nanograting and dielectric mirror. (c) Same as (b) but calculated at 895 meV resonance.

While anomalous Tamm plasmons have been predicted using Zak phase formalism,^23^ the latter offers little physical insight into the exact nature of such peculiar optical states. To understand it, we decided to rigorously model the interaction of the entire sample (i.e., nanograting residing on top of the dielectric mirror) with polarized light using a full-wave numerical solver implemented in COMSOL Multiphysics. The reflectivity spectra of the sample obtained numerically are shown in Figure 2c. Evidently, a good agreement with the experimental data is now achieved also for incident light polarized across the slits with a reflectivity dip emerging in the “forbidden” energy band, at 895 meV. The corresponding distribution of electric field intensity along the structure appears very similar to that calculated at the reflectivity dip for the other polarization, revealing an optical mode characteristic of a Tamm plasmon (see Figure 3a). The locations of both resonances are also consistent with the energies given by the phase matching condition in each case, where φ_DM_(*E*) and φ_nG_(*E*) were extracted directly from the simulated data. Importantly, while the φ_nG_(*E*) curve obtained numerically is identical to that calculated using the effective optical constants for light polarized along the slits, it appears quite different from what the effective medium approach predicted for the orthogonal polarization (compare dotted and solid blue curves in Figure 2b,d). More specifically, φ_nG_(*E*) now features a negative rather than positive slope and is offset within the photonic band gap by about +π/2, which reveals the true origin of the observed anomaly—for light polarized across the slits, the nanograting appears optically closer to the dielectric mirror than it physically is.

The explanation of such a peculiar “mirage” phenomenon is offered by Figure 3b,c, where we plotted and compare the distributions of the electric field amplitude near the surface of the gold nanograting for the two Tamm plasmon resonances. One can see that when the Tamm plasmon is excited by light polarized across the slits (at 895 meV), the nanograting strongly perturbs the near field expelling the regions of zero electric field from the slits into the dielectric mirror, to a depth of about 70 nm (as evident from Figure 3c). The resulting arch-like field distortions below the slits can be regarded as virtual metallic ridges (since the electric field vanishes there), which effectively extend the nanograting toward the dielectric mirror shrinking the intracavity spacing—just as real nanocorrugations do.^32^ Clearly, the topology of the near field in nanostructured metal films cannot be taken into account (let alone captured) by effective medium models and TMM, which, therefore, naturally fail to predict the appearance of Tamm plasmon states in the “forbidden” high-energy band.

We note that, in principle, conventional Tamm plasmon states can be pushed into the upper half of the photonic band gap in a modified Bragg reflector either by making the top high-index layer physically thinner than λ/4^2,20,25^ or by introducing an additional low-index layer.^17,33,34^ In those cases, however, the anomalous Tamm plasmon will be equally affected and still appear more “blue” with its energy exceeding the upper limit characteristic of the optical state under a semi-infinite metal layer.

**Figure 4:**
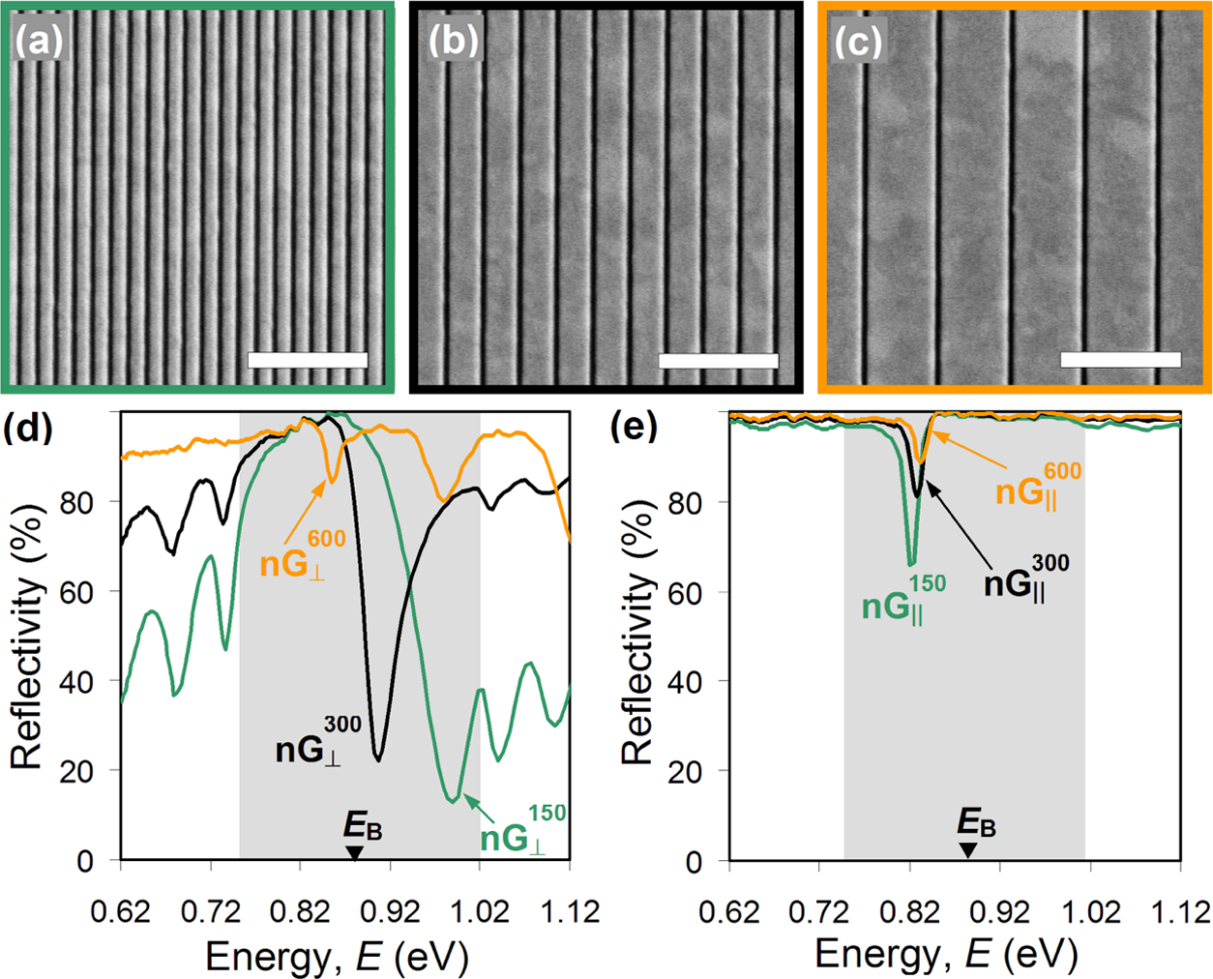
(a) SEM image of Au nanograting with the period of 150 nm fabricated on top of the dielectric mirror. Scale bar is 1 μm. (b) Same as (a), but the period is 300 nm. (c) Same as (a), but the period is 600 nm. (d) Reflectivity spectra of the nanogratings with the periods of 150 nm (green), 300 nm (black), and 600 nm (orange), respectively, measured for the polarization of incident light set orthogonal to the slits. The shaded area marks the location of the band gap of the dielectric mirror. (e) Same as (d), but the polarization of incident light is set parallel to the slits.

Given the microscopic origin of the high-energy Tamm plasmon, which involves the appearance of virtual corrugations below the nanograting, one may conclude that the energy level of this anomalous state must depend on the period of the nanograting. Indeed, for a smaller period, the corrugations would appear closer to each other effectively squeezing the electric field out of the gaps between them (and into the mirror) and, hence, should yield an even higher-energy Tamm state. Correspondingly, for a larger period, the virtual corrugations would be spaced further apart allowing the electric field to fill the gaps between them and extend further toward the nanograting. The energy of the resulting Tamm state should, therefore, approach that of a Tamm plasmon supported by the continuous Au film. This is exactly what we observed with further experiments involving two additional Au nanogratings (see Figure 4a,c). The latter also featured 50 nm wide slits but had periods of 150 and 600 nm, respectively, and were fabricated and characterized using the same procedures as described above for our first Au nanograting (Figure 4b).

**Figure 5:**
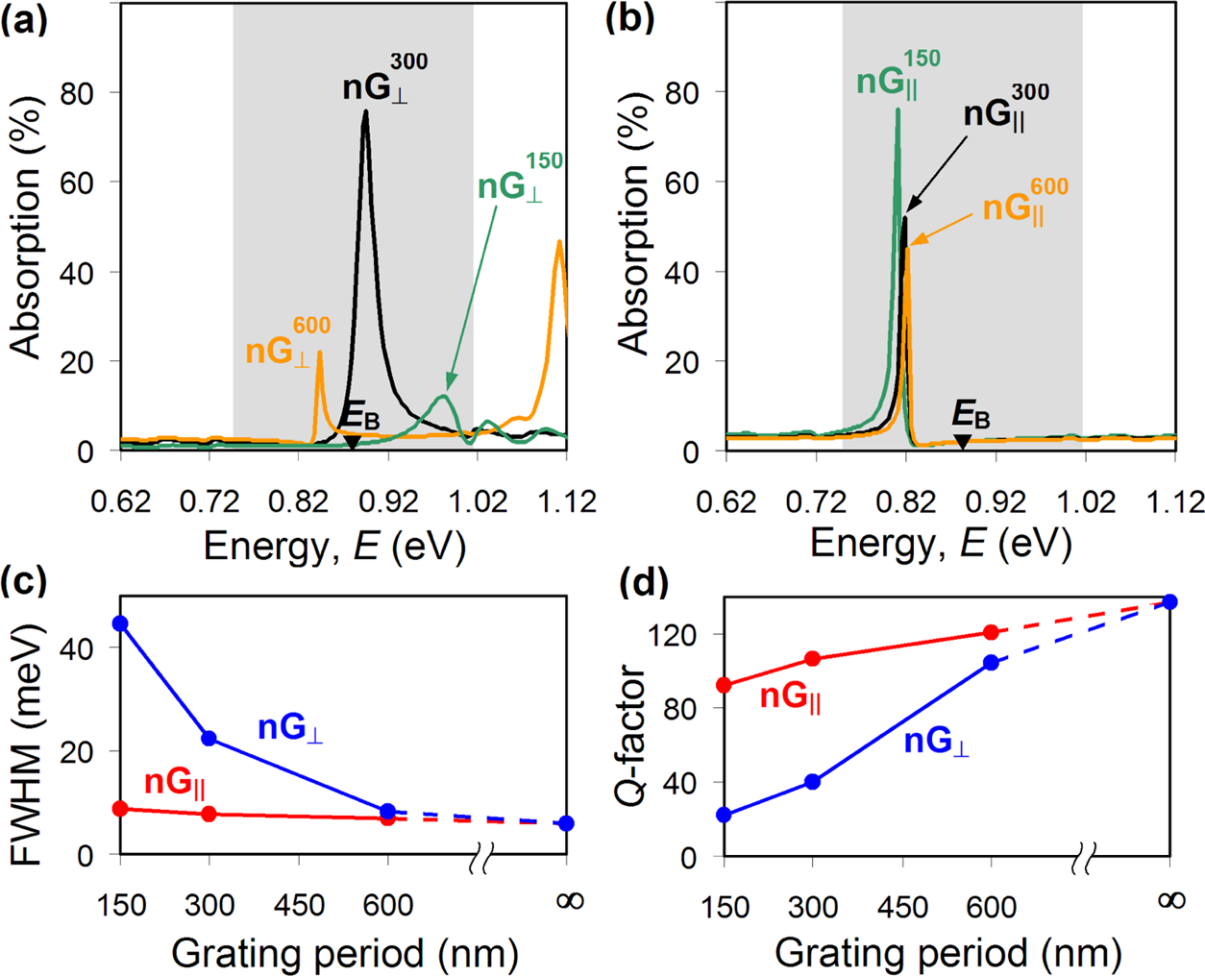
(a) Calculated absorption lines of Tamm plasmons excited under Au nanogratings with the periods of 150 nm (green), 300 nm (black), and 600 nm (orange), respectively, when the incident light is polarized orthogonal to the slits. The spectra were computed using a full-wave numerical model. The shaded area marks the location of the band gap of the dielectric mirror. (b) Same as (a), but incident light is polarized parallel to the slits. (c) Full width at half-maximum and (d) *Q*-factor of Tamm plasmon resonances plotted as functions of the nanograting period for incident light polarized parallel (red, nG_∥_) and orthogonal (blue, nG_⊥_) to the slits. The infinite period corresponds to unstructured Au film.

Figure 4d compares the reflectivity spectra of all three nanogratings measured for the polarization of incident light set across the slits. Clearly, an anomalous Tamm plasmon with the highest energy is excited under the nanograting with the smallest period. It corresponds to a reflectivity dip at 990 meV, right at the blue edge of the mirror’s photonic band gap. As the period of the nanograting increases from 150 to 600 nm, the energy of the anomalous state progressively decreases, reaching the level of 855 meV, still some 20 meV above the energy of a Tamm plasmon supported by the continuous Au film (see Figure 1b). For the incident polarization set long the slits, our data reveal the opposite trend, namely, the energy of a Tamm state decreases with decreasing period of the nanograting, although the changes are much smaller than in the previous case (see Figure 4e). Such trends are consistent with the fact that the volume fraction of Au in the nanograting becomes smaller as its period decreases, so optically it behaves as an Au film of a decreasing thickness.^20^ The spectral locations of the Tamm states that we observed experimentally with the shorter- and longer-period nanogratings also agree very well with the predictions of our full-wave numerical model (subject to 10 meV blueshift due to focused illumination), as evident from Figure 5a,b.

It is also instructive to analyze how the observed anomalous Tamm state evolves in terms of its linewidth and *Q*-factor and compare that with the case of a conventional Tamm plasmon resonance. Since the lifetimes of Tamm states observed in an experiment could be affected by the accuracy of employed fabrication and characterization techniques, we based our analysis on the simulated data presented in Figure 5a,b. The linewidth of Tamm resonances was calculated as the full width at half-maximum (FWHM) and is plotted in Figure 5c as a function of the nanograting period. One can see that with increasing period, the linewidth of the anomalous Tamm state decreases from 45 to about 7 meV, approaching the value of 6 meV attainable for a Tamm plasmon resonance in the case of a continuous 50 nm thick Au film. Expectedly, the *Q*-factor of the anomalous Tamm state exhibits the opposite behavior and is seen to increase from about 20 to 100, with the upper limit of 137 corresponding to the continuous Au film (Figure 5d). In fact, similar trends are also observed for the linewidth and *Q*-factor of a (conventional) Tamm plasmon excited under the nanograting when its slits are parallel to the polarization of incident light. Its resonances, however, have a smaller linewidth and, consequently, larger *Q*-factor. For example, the *Q*-factor of a Tamm plasmon state supported by the nanograting with a 300 nm long period appears to be almost 3 times larger.

The noted dependencies of the linewidth and *Q*-factor on the nanograting period can be readily anticipated by taking into account the leakage of light through the slits as an additional mechanism of radiative losses. Correspondingly, nanograting with a larger period has fewer slits and will, therefore, leak light at a slower rate (hence, larger *Q*). Furthermore, since the leakage of light through a subwavelength slit is much weaker when the latter is parallel to the electric field, Tamm plasmon resonances will generally be narrower and characterized by a larger *Q*-factor for light polarization set parallel to the slits of the nanograting.

## Conclusions

We observed experimentally and analyzed numerically the appearance of an anomalous near-IR Tamm plasmon state, which emerged at the interface between a conventional, completely periodic Bragg reflector and a subwavelength nonresonant gold nanograting. The observed state had the energy exceeding the theoretical limit achieved in the case of a continuous semi-infinite gold layer and could not be predicted in the frame of the effective medium approach and transfer matrix method routinely employed in the analysis of Tamm plasmons. We have found that the anomaly stemmed from a nontrivial topology of the near field produced by the nanograting inside the Bragg reflector. Our findings illustrate how the effective medium approach may have limited applicability even in the case of nonresonant nanostructured metal films featuring the most primitive structural patterns. The demonstration of the effect introduces a new practical degree of freedom in controlling the spatial localization of Tamm plasmons and enables attaining the energies of optical Tamm states across the entire photonic band gap with minimal structural alterations. Our findings also offer new insights into the design of hybrid photonic cavities, identifying an important additional degree of freedom in controlling their optical length.

Among the immediate applications of the observed anomalous Tamm state is polarization discrimination in reflection. Indeed, periodic 1D nanopatterning of Au film rendered the metal-coated Bragg reflector as an efficient polarization-sensitive mirror, which operated at normal incidence and had the extinction ratio of 1:10 at the wavelength of anomalous Tamm plasmon resonance. Carving nanogratings in the metal coating also allows one to easily adjust the energy of Tamm state within a wide spectral range and, therefore, constitutes a practically viable approach to tuning Tamm plasmons after fabrication. Moreover, the demonstrated approach enables spatial modulation of the energy of Tamm state along a metal-coated Bragg reflector, whereby an array of Tamm plasmon-based sensors or light emitters can be engineered on the same chip, all operating at different wavelengths.

## Supporting Information Available

Schematic of the fabricated structure and reflection and transmission of the nanograting modeled using effective medium approach and full-wave simulation (PDF)
