# Supplementary material for: Observation of a High-Energy Tamm Plasmon State in the Near-IR Region
Source: ACS Appl Mater Interfaces. 2022 Mar 9;14(11):13638–44. doi: 10.1021/acsami.2c00486 (PMC9096782; doi:10.1021/acsami.2c00486)
Supplement: Supplementary file 1 — am2c00486_si_001.pdf [file am2c00486_si_001.pdf]

Supporting Information

Observation of a High-Energy Tamm Plasmon State in the Near-IR

Oleksandr Buchnev,[1,2] Alexandr Belosludtsev,[3] and Vassili A. Fedotov[1,2,*]
[1]*Optoelectronics Research Centre, University of Southampton, Southampton SO17 1BJ, UK*
[2]*Centre for Photonic Metamaterials, University of Southampton, Southampton SO17 1BJ, UK*
[3]*Optical Coating Laboratory, Center for Physical Sciences and Technology, Vilnius LT-02300, Lithuania*

**email: vaf@orc.soton.ac.uk*

**Bragg Reflector-Based Hybrid Structure
Designed to Support Tamm Plasmons in Near-IR**

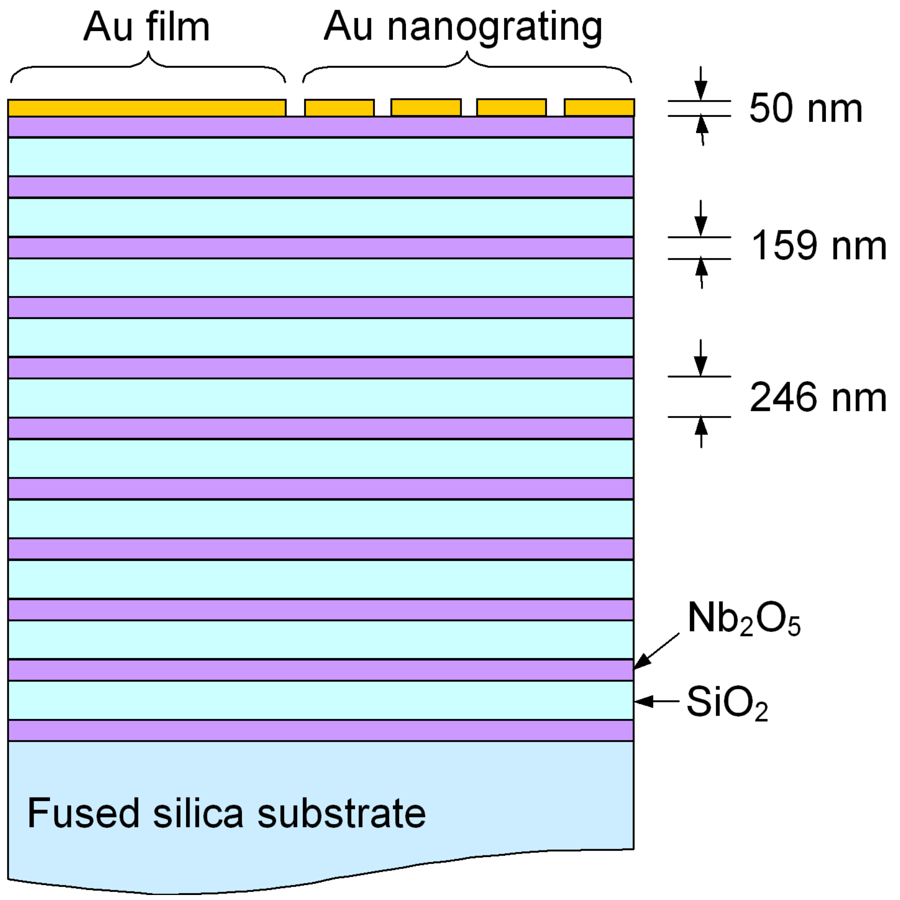

Figure S1. Schematic of the fabricated sample: 11 layers of Nb_2O_5 and 10 layers of SiO_2 deposited on a fused silica substrate and capped by a thin gold film, a part of which is patterned in the form of a non-diffracting 1D nanograting.

Modeling Optical Properties of Nanograting: Effective Medium Approach vs. Full-Wave Simulation

We extracted the effective optical constants of the nanograting using the following procedure. First, we calculated the transmission and reflection of the nanograting, T_{grat} and R_{grat}, numerically, using commercial simulation software COMSOL Multiphysics. The simulations took into account the effect of the mirror's top high-index layer (i.e., Nb_2O_5), which acted as a substrate and was extended to infinity to simplify the extraction of the effective optical constants later on. In the simulations, the optical constants of Au were defined by the tabulated data;[1] the refractive index of Nb_2O_5, n_{sub}, was assumed to be constant across the entire energy band and equal to 2.24.[2] Next, given the sub-wavelength period of the nanograting we assumed that its optical response could be mimicked by a continuous film of some effective material of the same thickness, d, described by the optical constants n and k. The transmission and reflection coefficients of such a thin film resting on a substrate, T_{film} and R_{film}, would then be given by the following formulae:[3]

$$T_{film} = \left| \frac{\dfrac{4}{1+n-ik} \cdot \dfrac{n-ik}{n-ik+n_{sub}} \cdot e^{-\frac{2\pi k}{\lambda} d}}{1 + \dfrac{1-n+ik}{1+n-ik} \cdot \dfrac{n-ik-n_{sub}}{n-ik+n_{sub}} \cdot e^{-\frac{4\pi k}{\lambda} d}} \right|^2 n_{sub} \quad \text{(S1)}$$

$$R_{film} = \left| \frac{\dfrac{1-n+ik}{1+n-ik} + \dfrac{n-ik-n_{sub}}{n-ik+n_{sub}} \cdot e^{-\frac{4\pi k}{\lambda} d}}{1 + \dfrac{1-n+ik}{1+n-ik} \cdot \dfrac{n-ik-n_{sub}}{n-ik+n_{sub}} \cdot e^{-\frac{4\pi k}{\lambda} d}} \right|^2 \quad \text{(S2)}$$

Rather than trying to solve directly the above system of equations for $T_{film} = T_{grat}$ and $R_{film} = R_{grat}$, we employed the Monte Carlo approach, whereby we randomly generated values of n and k in the range 0 – 14 (as defined by min and max values of the optical constants of bulk Au) and using Eq. S1 selected only those pairs of n and k that would yield $|T_{film} - T_{grat}| < 0.1$ %. Then, following the same approach but using Eq. S2 we selected a second set of random pairs of n and k that would yield $|R_{film} - R_{grat}| < 0.1$ %. In (n, k) space each of those sets of points traced out a smooth curve. The two curves crossed one another and their intersection marked the approximate solution of the system of equations (S1) and (S2), which therefore gave the effective optical constants of the nanograting.

Figures S2 shows the effective optical constants of the nanograting for the two polarizations of incident light (i.e., parallel and orthogonal to the slits) and compares them with the optical constants of bulk Au. Clearly, in both cases the nanograting is seen to behave as a film of some plasmonic metal, which is expected given the large volume fraction of Au in the structure. The extracted optical constants enabled us to reproduce, using Eqs. (S1) and (S2), the simulated transmission and reflection of the nanograting with very high accuracy (exceeding 0.3 %), as illustrated by Fig. S3.

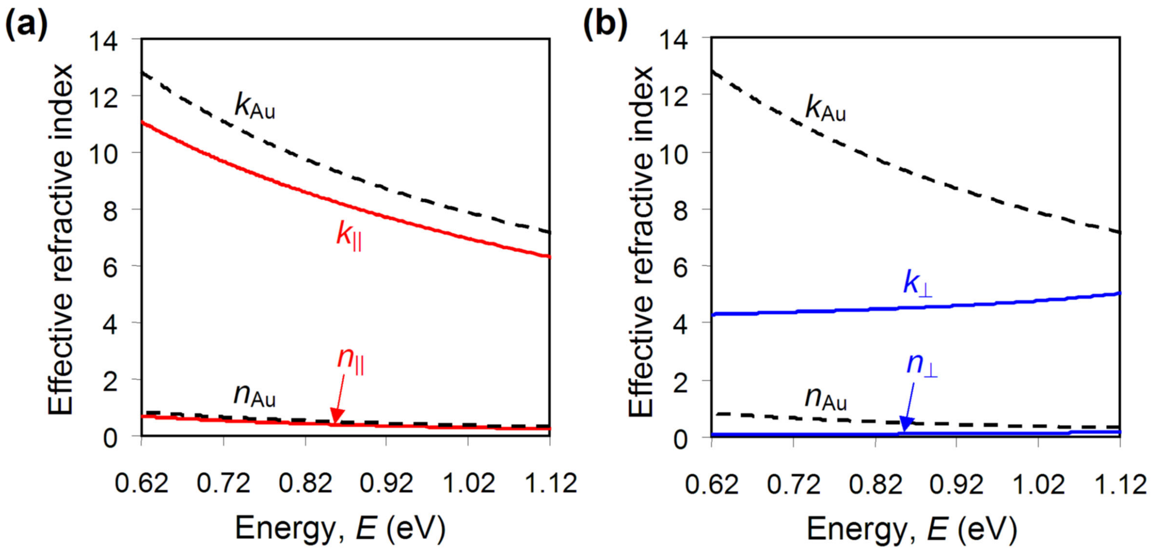

Figure S2. Dispersion of the effective complex refractive index of the nanograting calculated using the effective medium approach for light polarised along (a) and across (b) the slits of the nanograting. Black dashed curves represent the reference case of bulk gold.[1]

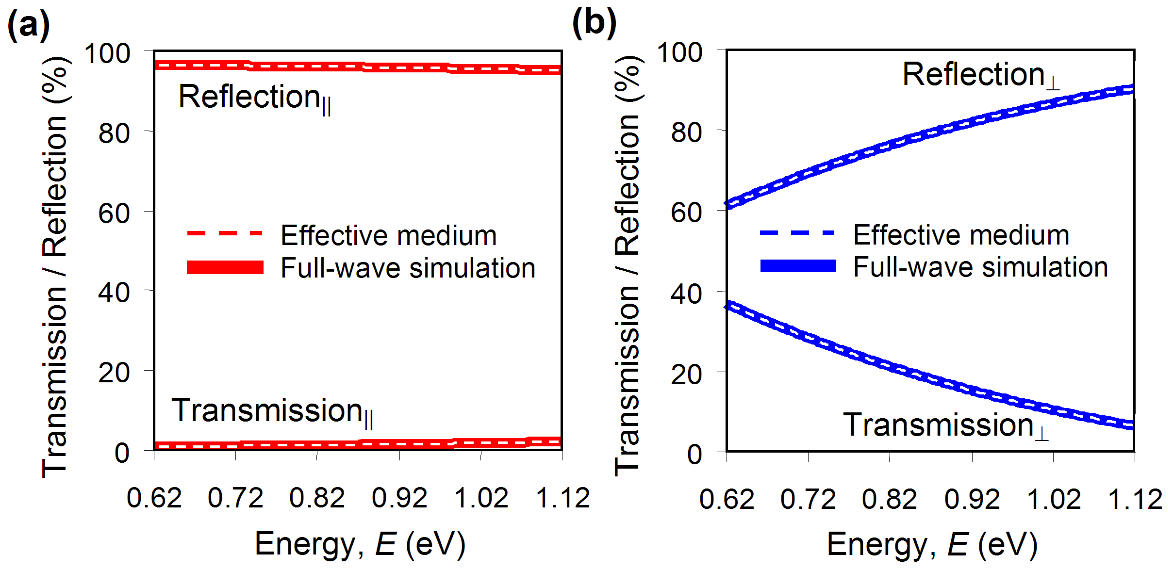

Figure S3. Transmission and reflection spectra of the nanograting calculated using the effective medium approach and full-wave simulation for light polarised along (a) and across (b) the slits of the nanograting.

References

[1] Palik, E. D., *Handbook of Optical Constants of Solids* (Academic Press, London, 1985).

[2] Juškevičius, K., Audronis, M., Subačius, A., Kičas, S., Tolenis, T., Buzelis, R., Drazdys, R., Gaspariūnas, M., Kovalevskij, V., Matthews, A., Leyland, A., "Fabrication of Nb_2O_5/SiO_2 Mixed Oxides by Reactive Magnetron Co-Sputtering," Thin Solid Film 589, 95-104 (2015).

[3] Heavens, O. S., *Optical Properties of Thin Solid Films* (Butterworths Scientific Publications, London, 1955).
